# Diagnosis of Pulmonary Infections Due to Endemic Fungi

**DOI:** 10.3390/diagnostics11050856

**Published:** 2021-05-10

**Authors:** Victoria Poplin, Clarissa Smith, Dominique Milsap, Lauren Zabel, Nathan C. Bahr

**Affiliations:** 1 Department of Internal Medicine, Division of Infectious Diseases, University of Kansas, Kansas City, KS 66160, USA; vpoplin@kumc.edu; 2 Department of Internal Medicine, University of Kansas, Kansas City, KS 66160, USA; csmith12@kumc.edu; 3 School of Medicine, University of Kansas, Kansas City, KS 66160, USA; d883m097@kumc.edu (D.M.); lzabel@kumc.edu (L.Z.)

**Keywords:** endemic fungi, diagnostic tests, histoplasmosis, blastomycosis, coccidioidomycosis, paracoccidioidomycosis, talaromycosis, pulmonary infection

## Abstract

Endemic mycoses including *Histoplasma*, *Blastomyces*, *Coccidioides*, *Paracoccidioides*, and *Talaromyces* are dimorphic fungi that can cause a variety of clinical manifestations, including respiratory infections. Their pulmonary presentations are variable, and diagnosis is often delayed as they can mimic other infectious and non-infectious causes of pulmonary disease. Delay in diagnosis can lead to unnecessary antibiotic use, repeat hospitalizations, and increased morbidity and mortality. The diagnosis of endemic fungal pulmonary infections often relies on multiple diagnostic tests including culture, tissue histopathology, antigen assays, and antibody assays. Due to the increased use of immunosuppressive agents and the widening geographic ranges where these infections are being found, the prevalence of endemic fungal infections is increasing. Physicians need to be aware of the clinical manifestations of pulmonary infections due to endemic fungal in order to ensure that the proper diagnostic work up is obtained promptly. A high index of suspicion is particularly important in patients with suspected pulmonary infections who have failed to improve despite antibiotics in the appropriate setting. We present a review diagnostic testing for pulmonary infections due to endemic mycoses.

## 1. Introduction

Fungal pneumonia caused by endemic fungi including *Histoplasma*, *Blastomyces*, *Coccidioides*, *Paracoccidioides,* and *Talaromyces* can be challenging to diagnose. These mycoses are termed endemic as they classically occur in particular geographic regions [1]. Endemic fungi are dimorphic, existing as molds at cooler environmental temperatures and yeast within the warmer temperatures of the human body. The endemic fungi can cause a variety of syndromes, but all carry the potential to cause respiratory infections since inhalation is a major mode of disease acquisition in humans. Furthermore, rates of pneumonia due to endemic fungi are increasing due to increased use of immunosuppressive therapies [2]. Diagnosis of pneumonia due to endemic fungi can be challenging as the clinical presentations are varied and non-specific, meaning this type of pneumonia may be mistaken for a variety of infectious or non-infectious causes of lung disease [1,3]. The diagnosis is frequently delayed, particularly when occurring outside of traditional endemic areas as physicians may not be familiar with the disease manifestations [1].

Multiple diagnostic tests may be required for diagnosis including tissue histopathology, culture, or specific fungal antigen or antibody detection assays in the appropriate clinical scenario. One non-specific fungal diagnostic test may be useful in the diagnosis of *Coccidioides*, *Histoplasma*, *Talaromyces*, and *Paracoccidioides* is detection of 1,3-β-d-glucan, a component of the cell wall of many fungi [4,5,6]. The 1,3-β-d-glucan assay is less useful to detect *Blastomyces* given its yeast phase produces low levels of 1,3-β-d-glucan [4]. 1,3-β-d-glucan assay is a nonspecific test, therefore additional testing is needed to differentiate between fungi when the test is positive. Accordingly, the main role for 1,3-β-d-glucan testing in the diagnosis of endemic fungal pneumonia is as a screening test, or a non-specific test to help one further narrow the planned workup. We present a review of diagnostic testing for pneumonia due to endemic fungi. Given that geographic location and clinical presentations are crucial to proper diagnosis, these factors are described as well.

## 2. Histoplasmosis

Histoplasmosis is caused by *Histoplasma capsulatum var capsulatum* and *Histoplasma capsulatum var. duboisii* [7]. Classically, *H capsulatum* is thought of as endemic to the Ohio and Mississippi River Valleys in the United States, as well as parts of Central and South America [2,7,8,9,10]. More recently it has become clear that *Histoplasma* occurs frequently in many parts of the world including: Central and Eastern North America, the majority of Central and South America, much of sub-Saharan Africa, large portions of southeast Asia and small areas within Australia and Europe [7]. *H duboisii* has primarily been described in West Africa.

Infection is acquired through inhalation of spores from soil that is contaminated with bird or bat droppings [9,10]. *Histoplasma* can cause a wide variety of clinical manifestations including a spectrum of pulmonary diseases ranging from acute to chronic presentations [9,11]. Table 1 describes signs, symptoms, imaging and lab findings, and epidemiology of histoplasmosis and other endemic fungi.

Acute pulmonary histoplasmosis (APH) typically presents with fever, chills, shortness of breath, and resembles community acquired pneumonia [2,3,9,11,12]. APH can range from a mild self-limiting illness to acute respiratory distress syndrome [2,9,11,13]. Subacute pulmonary histoplasmosis (SPH) has a more insidious onset over at least one month and may develop after a smaller inoculum exposure [2,3,9,11]. Chronic pulmonary histoplasmosis (CPH) is classically seen in older males with underlying lung disease [2,3,10,11]. CPH has a similar presentation to tuberculosis with fever, night sweats, weight loss, cough, and dyspnea over at least three months [9,10,11,12]. *H capsulatum* may also cause pulmonary nodules, mediastinal adenitis, mediastinal granulomas, and mediastinal fibrosis [1,2]. Progressive disseminated histoplasmosis is a form of histoplasmosis that result from hematogenous spread and can impact multiple organ symptoms including the respiratory tract and cause severe disease [11].

In APH, imaging frequently shows diffuse bilateral patchy opacities with hilar and mediastinal adenopathy while diffuse reticulonodular or miliary infiltrates can be seen less commonly [2,3,11,13,14]. In CPH patchy infiltrates can progress to large, destructive cavities; hilar and mediastinal lymphadenopathy are uncommon compared to APH [10,11].

**Figure 1:**
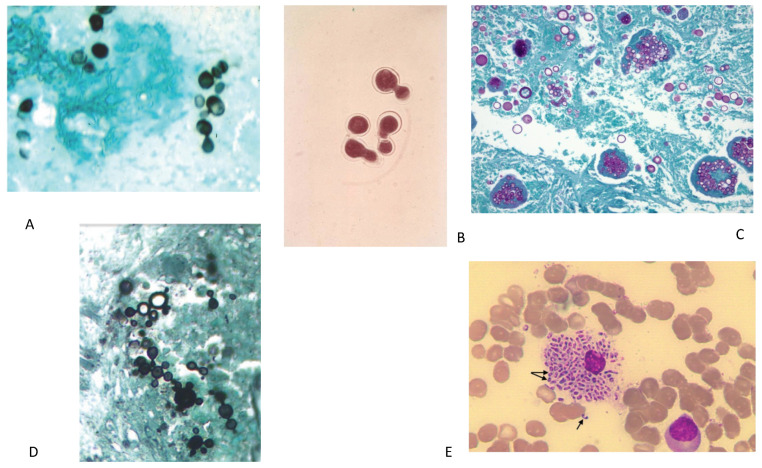
(**A**) Gomori methenamine silver stain showing small oval budding yeast form of *H. capsulatum* yeast. (**B**) KOH wet mount showing *B. dermatitidis* yeast with characteristic broad-based budding. (**C**) PAS stain *C. immitis/posadasii* endospore containing spherules with round thick walls in the tissue. Figure 1A–C from Wheat LJ, Goldman M, Hage CA, Knox KS, Cryptococcosis and the Endemic Mycoses In: Grippi MA, Elias JA, Fishman JA, Kotloff RM, Pack AI, Senior RM, Siegel MD, *Fishman’s Pulmonary Diseases and Disorders*. 5th ed. McGraw-Hill Education: 2015. Figure 134-3 [131]. (**D**) Typical multi-budding yeast cells (black staining) with a ‘ships-wheel’ appearance in a tissue sample of *Paracoccidioides* (Grocott-Gomori methenamine silver). Figure 1D from Restrepo-Moreno A, Tobόn-Orozco, AM, González-Marín A, Paracoccidioidomycosis. In: Bennett JE, Dolin R, Blaser MJ, *Mandell*, *Douglas and Bennett’s Principles and Practice of Infectious Diseases*. 9th ed. Elsevier: 2020. Figure 267.6B [132]. (**E**) Wright’s stain of bone marrow aspirate of patient showing numerous small, non-budding oval yeast cells measuring 5–6 um inside an engorged histiocytes. The arrows show the actively dividing yeast cells, revealing a midline septum characteristic of *Talaromyces marneffei*. Figure 1E from Trieu Ly V, Tat Than N, Chan J, Day JN, Perfect J, Ngoc Nga, C, Van Vinh Chau N, Le T. Occult Talaromyces marneffei Infection Unveiled by the Novel Mp1P Antigen Detection Assay. *Open Forum Infectious Diseases*. 2020. (PMID 33269295) [124].

Identification of *H capsulatum* on histopathology and culture is the classical diagnostic standard [2,10,15]. The narrow based budding ovoid *Histoplasma* yeast (2–4 μM in diameter) is visualized via direct microscopic examination or the use of Gomori methenamine silver, Giemsa, periodic acid-Schiff, or hematoxylin eosin stains of specimens such as respiratory samples, lymph node tissue, or lung tissue (Figure 1A) [1,2,8,9,11,16,17,18]. Cytopathologic examination of bronchoalveolar lavage (BAL) fluid is positive in up to 50% of cases [2,3,8]. Histopathologic examination can reveal both caseating and non-caseating granulomas [8,9]. Despite the potential to improve diagnosis, pathological examination is not feasible in most patients as it requires invasive procedures, such as bronchoscopy or biopsies [8]. In general, it is more useful in disseminated histoplasmosis compared to pulmonary histoplasmosis and is more likely to be positive in SPH or CPH compared to APH [11,19]. *Histoplasma* can take 2–8 weeks to grow on culture which is similarly more likely to be positive in SPH or CPH compared to APH [2,3,8,9,10,16,19]. Overall sensitivity of culture of sputum or bronchoscopy specimens is 48–75% in pulmonary histoplasmosis [3,11]. Table 2 shows the performance of various tests for histoplasmosis and the other endemic fungi.

Antigen detection can provide rapid diagnosis of pulmonary histoplasmosis. Numerous commercial and in-house tests are available, however, agreement between tests is not uniform [12,15,24]. Most though not all antigen tests use an enzyme immunoassay (EIA) [12,15,21]. Antigen is generally more likely to be positive in APH compared with SPH and CPH although CPH commonly yields positive results as well [2,3,11,19]. In a multicenter evaluation by Hage et al., antigenuria was detected in 83% of acute cases, 30% of subacute cases, and 87.5% of chronic pulmonary histoplasmosis, with the highest antigen concentrations in acute cases—combined urine and serum antigen testing improved yield [19]. In another large study of APH, antigen was detected in serum and urine in 65% and 69% of cases, respectively [13]. This same study found that antigen was more likely to be detected in patients who require hospitalization, likely reflecting higher fungal burden in more severe disease [13]. Antigen testing of BAL can further aid in diagnosis of pulmonary histoplasmosis, particularly in CPH or diffuse pulmonary disease complicating disseminated histoplasmosis. Hage et al. found that among 31 patients with histoplasmosis and pulmonary involvement, antigen detection in BAL had 93.5% sensitivity, 97.8% specificity, 68.8% positive predictive value, and 99.6% negative predictive value [20]. Overall, 21 of the 31 patients in this study were immunocompromised with disseminated histoplasmosis including disseminated pulmonary disease [20]. One limitation of *Histoplasma* antigen testing is its cross reactivity with other mycoses, such as *Blastomyces* spp, *Talaromyces marneffei*, *Paracoccidioides*, *Coccidioides*, and *Aspergillus* spp. [2,3,10,11,12,14,15,19,21,22,24].

The IMMY *Histoplasma* EIA, is a commercially available, FDA approved EIA for detection of *Histoplasma* antigen in urine [12,15]. In a study by Theel et al. the IMMY EIA had 97.6% agreement with MiraVista Diagnostic’s EIA and a specificity and sensitivity of 99.8% and 64.5%, respectively [15]. As opposed to MiraVista’s EIA which is done at a central laboratory, health centers can perform IMMY’s test given its FDA approval. MiraVista recently developed a lateral flow assay (LFA) for serum antigen detection [66]. In patients with HIV and disseminated histoplasmosis, sensitivity was 96% and specificity 94% [66]. This test is not FDA approved.

Antibody testing is also used to diagnose histoplasmosis. Because antibodies take time to develop after acute infection, they are more useful in SPH and CPH than APH and negative initial antibody testing should be repeated in one to two months if suspicion is high [2,3,8,9,10,11,13,14,17]. Additionally, antibody testing may be negative in immunocompromised patients and may cross react with other endemic mycoses such as *Blastomyces*, *Paracoccidioides*, and *Coccidioides* [2,8,9,11]. Common methodologies include immunodiffusion (ID), complement fixation (CF), or EIA [2,8,9,11]. ID detects H and M bands, H bands are more rare but when found indicate acute infection whereas M bands are more common and may persist for years [2,8,11]. A fourfold rise in CF titers or a single titer of 1:32 or higher is indicative of active infection [2,8,11]. Compared to CF, ID is slightly more specific and less sensitive [10]. One multicenter evaluation found 67% seropositivity (by CF or ID) in APH, compared to 95% in SPH and 83% in CPH [19]. In one study of patients with APH, MiraVista Diagnostics’ IgM, and IgG EIA exhibited 89% sensitivity and 92% specificity [14]. Combining antigen and antibody testing may improve sensitivity for diagnosis of APH, potentially as high as 96% [13,14]. Similar sensitivity has been found using the combination of BAL antigen detection and BAL cytopathology [3].

Nucleic acid amplification tests (NAAT) such as polymerase chain reaction (PCR) or loop-mediated isothermal amplification (LAMP) have been utilized for the identification of *H. capsulatum*, however these have variable sensitivities and none are commercially available [2,67,68,69,70,71,72,73,74]. NAATs are less likely to have false positive results due to other endemic fungi compared to antigen and antibody testing [67]. A reference database has been created to identify *H. capsulatum* via matrix assisted laser desorption ionization-time of flight mass spectrometry (MALDI-TOF MS) but little data on performance is available [75,76]. Similarly, while metagenomic next generation sequencing (mNGS) has been used on BAL fluid to diagnose *H. capsulatum* causing chronic progressive pulmonary lesions and epiglottis lesions, broader performance data are not available [77,78]. Finally, there may be a role for panfungal PCR to diagnose histoplasmosis, but so far use has been exploratory [79].

Pulmonary infection is more common due to *H. capuslatum var capsulatum* than *H. capsulatum var. duboisii* [7]. In a summary of 94 reported cases, only 10 were suspected to have pulmonary involvement [80]. *H capsulatum var. duboisii* frequently causes lymphadenopathy, bone, cutaneous, sub-cutaneous, and disseminated disease, and may occur decades after leaving the endemic area [80]. *H capsulatum var. duboisii* diagnosis has not been well studied and so diagnostic test performance characteristics are less well understood. Histopathology, cytology are commonly used while confirmation with culture or PCR are less common and antibody testing is even more rare [80]. Histoplasma antigen testing has not been utilized [80].

## 3. Blastomycosis

*Blastomyces dermatitidis*, *Blastomyces gilchristii* and the recently characterized *Blastomyces helicus* and *percursus* can cause pneumonia in both immunocompetent and immunocompromised individuals [33,81,82,83]. *Blastomyces* spp are likely endemic throughout the midwestern United States and eastern North America, much of Africa and the majority of India [7,25,33,81,82,84]. Blastomycosis occurs due to inhalation of *Blastomyces* spores commonly found in sandy soils with an acidic pH, near a water source within forested areas with vegetative decay [1,25,33,81]. Activities associated with infection include landscaping, construction, fishing, and hunting [26,33,85]. *Blastomyces* infection causes nonspecific symptoms and diagnosis is frequently delayed, leading to excess healthcare visits and courses of antibiotics [25,33]. Pulmonary infection due to *Blastomyces* may cause mild or severe acute infections including acute respiratory distress syndrome, and chronic presentations that mimic tuberculosis or malignancy [1,25,28,33,83,84].

Definitive diagnosis of pulmonary blastomycosis is achieved by identification of *Blastomyces* through direct visualization of yeast or growth on culture. Staining of sputum with potassium hydroxide, calcofluor white, Gomori methenamine silver or periodic acid-schiff can help visualize the yeast form of *Blastomyces* [25,26,33,83]. *Blastomyces* yeast is 8–20 μM with broad based budding and a doubly refractile cell wall (Figure 1B) [30,33,34]. Direct identification of *Blastomyces* in clinical specimens can lead to diagnosis rapidly and before culture or other testing methods [25,33,83]. The potassium hydroxide smear is an appealing test as it takes only 15–30 min, but its sensitivity varies from 50–90% [25]. Fungal culture is the gold standard for diagnosing *Blastomyces* as it is highly specific, though growth typically 5–14 days and may take up to five weeks [25,28,33,34,83]. In one study, culture from sputum, tracheal secretions, or bronchial washings had sensitivities of 75%, 100%, and 67%, respectively, while combining results from multiple sources improved yield [28].

EIA detection of *Blastomyces* galactomannan antigen is also utilized [25,29]. Urine antigen testing has a sensitivity of up to 93% and specificity of 79% in pulmonary and extrapulmonary blastomycosis and serial urine antigen measurements are sometimes used to monitor treatment response [25,27,29,31,32,33]. Serum antigen testing can be done but sensitivity is sub-optimal (57% even with EDTA-heat treatment, improved from 36% without) however BAL fluid antigen testing sensitivity may be as high as 82% in pulmonary blastomycosis [3,26,29,31,32]. Limitations of antigen testing include delayed results due to transit times required for shipping to reference laboratories and cross-reactivity with other endemic fungi such as *Histoplasma*, *Paracoccidioides*, and *Talaromyces* [1,25,27,31,32,33,34].

CF and ID testing for *Blastomyces* have variable reported sensitivity and specificity [28,33,34,35,36]. A more sensitive (88%) EIA that detects antibody against the BAD1 protein also has higher specificity (94–99%) but is not commercially available [36]. As is typical for antibody testing in infectious diseases, utility is lower in early disease prior to antibody production and in some immune suppressed patients due to inability to produce antibodies [33,36]. PCR assays have been developed but are not commercially available and may be limited by polymorphisms within target regions [86,87,88]. Interestingly, one real time PCR test has been designed to test for both *H. Capsulatum* and *B. dermatitidis* with a sensitivity of 86% and specificity of 99% for *B. dermatitidis* [67]. mNGS has been used to diagnosis blastomycosis from a transbronchial biopsy and BAL fluid but diagnostic performance characteristics are unknown [89]. Whole genome sequencing may have a role for identification, but experience is limited at this time [90].

## 4. Coccidiomycosis

Coccidiomycosis colloquially known as “Valley Fever” is caused by *Coccidioides immitis* and *Coccidioides posadasii* [44,45,91,92,93]. *C. immitis* predominates in central California but has been found as far north as Washington, whereas *C. posadasii* is the predominant species in South and Central America, Mexico, Arizona, Texas, and Utah [7,44,91,92,94]. The primary route of infection is via inhalation of arthroconidia [40,44,45,91]. Many individuals who are exposed do not develop clinical symptoms [41,44,45,92,95]. The most common presentation is acute pulmonary infection similar to community acquired pneumonia with fever, headache, cough, fatigue, and pleuritic chest pain among the common symptoms though presentations may be more severe in immune compromised persons [40,41,45,93,96]. Additionally, *Coccidioides* more frequently causes associated hilar and paratracheal adenopathy on imaging compared to bacterial pneumonia and may cause chronic progressive cavitary lesions or pulmonary nodules [40,41,45,96].

The standard for diagnosis of coccidioidomycosis is culture or identification of spherules on histological examination of clinical specimens [40,45,91,96]. Spherules range in diameter from 10 to 200 μm, are filled with 2–5 μm diameter endospores and can be visualized with Papanicolaou, calcofluor white stain, potassium hydroxide, periodic acid Schiff, Grocott-methenamine silver, or hematoxylin-eosin stains (Figure 1C) [40,41,43,45,97]. Clinical specimens may lack intact spherules and instead have numerous endospores which can be mistaken for *Histoplasma*, *Blastomyces,* or *Cryptococcus* [91]. *Coccidioides* can typically be grown on routine culture in one week where a chemiluminescent DNA probe (Accuprobe, GenProbe, San Diego, CA, USA) can then be used for rapid identification, however, the laboratory should be informed of suspicion for coccidioidomycosis prior to sending the specimen given that arthroconidia are easily aerosolized [40,41,42,43,97].

Serologic testing via tube precipitin (TP) and CF are commonly used due to ease of specimen collection and laboratory safety [40,45,91,97,98]. TP tests for IgM, whereas CF tests for IgG [91]. Immunodiffusion (ID) can also be used to test for both IgM and IgG and may be referred to as IDTP and IDCF as they use the same antigen preparations as TP and CF [91,97,98]. CF and ID are often only available at reference laboratories [45,46]. Commercial EIAs for both IgG and IgM are now available, are more sensitive than traditional antibody tests, and can detect antibody earlier in the disease course [40,44,45,47,48,91,99]. A positive IgG on EIA should be sent to a reference lab for confirmation and quantification with CF [40,45,97]. An isolated positive EIA IgM should be confirmed with repeat EIA testing or another method as false positive results have been noted duet to blastomycosis and other fungal infections [43,46,47,49,97]. CF titers can be utilized to monitor treatment response and can provide prognostic information [41,96]. As with other antibody tests, those for *Coccidioides* may be negative early in disease (repeat testing should be done if suspicion is high) and may be falsely negative in immune suppressed patients; combining antibody tests may improve sensitivity [37,41,42,45,46,91,96,97].

An EIA for detection of *Coccidioides* antigen in urine and serum is now commercially available [39,40,41,42,91]. One study found 71% sensitivity using urine antigen testing in severe disease in a mostly immunocompromised population–further study is needed in isolated pulmonary diseases where one would expect relatively decreased sensitivity [42]. Skin testing for Coccidioides was available until the late 1990s, in 2011 a newly formulated skin test (Spherusol, Nielson Biosciences, San Diego California) was approved by the FDA [100]. Spherusol can be utilized to test for cellular immunity to Coccidioides due to prior infection but its role in diagnosis of active infection is uncertain [50,100]. Coccidioides specific PCR is infrequently used for diagnosis and panfungal PCR’s role is exploratory at best at this point while use of MALDI-TOF MS for identification of coccidioidomycosis is similarly rare although commercial MALDI-TOF MS panels are now available for *Coccidioides immitis* and *C posadasii* [79,101,102,103,104]. Whole genome sequencing may also be an option for identification of *Coccidioides,* particularly for cluster investigations [105].

## 5. Paracoccidiomycosis

Paracoccidiomycosis is caused by *Paracoccidioides brasiliensis* or *Paracoccidioides lutzii* and is endemic in parts of South, Central, and North America, but is most common in Brazil [7,52,54,55,106]. *Paracoccidioides lutzii* has only been reported in limited regions of Brazil fairly recently while *P. braziliensis* has been reported in Argentina, Venezuela, and Mexico as well—most descriptions of clinical disease refer to *P braziliensis* [7]. *Paracoccidioides* infection is associated with exposure to contaminated soil and occurs after inhalation of fungal conidia [54,56,106,107]. *Paracoccidioides* can cause infection in both immunocompromised and immunocompetent individuals [54]. The acute or subacute form is typically seen in children or adults less than 30 years old and may cause fever, weight loss, and lymphadenopathy. Disseminated disease commonly includes gastrointestinal, cutaneous, or osteoarticular involvement [52,54,55,106]. Pulmonary disease is rare and is more common in the common (adult) form which occurs due to reactivation or reinfection and causes symptoms similar to tuberculosis (cough, dyspnea, weight loss, anorexia) [54,55,106]. In patients with HIV, paracoccidioidomycosis progresses more rapidly and is more likely to disseminate [54,55,106]. A relatively large descriptive study of *P. lutzii* cases describes 34 cases, all of the ‘chronic’ or adult form of which 28 (82.4%) had pulmonary involvement [108].

Traditionally diagnosis is achieved by identification of the characteristic yeast form in tissue or clinical specimens [51,52,54,56,106,107]. The yeast are large mother cells surrounded by multiple narrow-necked budding daughter cells resembling a “pilot wheel” or mother cells with only two daughter cells resembling a “Mickey mouse head” (Figure 1D) [52,106,107]. In one study, cytopathological examination on sputum had sensitivities of ~60% for both acute and subacute and chronic paracoccidiomycosis [53]. Culture is also possible, however less clinically useful given culture growth takes 2–4 weeks [55,106,107]. Typical methods of identification in clinical specimens or culture are not able to differentiate *P. brasiliensis* from *P. lutzii,* at this point genotyping is required to do so [108].

Antibody detection through double immunodiffusion (DID), immunoblots (IB), latex agglutination (LA), counterimmunoelectrophoresis (CIE), and enzyme linked immunosorbent assay (ELISA) is available at reference laboratories for diagnosis of *P brasiliensis*, with DID being utilized most frequently [52,53,54,56,57,107]. The sensitivity and specificity of DID is high, 90% and 100%, respectively, in one study [53]. DID titers may be used to monitor response to treatment with higher levels typical in acute and subacute and disseminated forms [52,53,54,106]. Antibody testing, particularly ELISA may cross-react with *Histoplasma* and other fungi [52,56,58,106,107]. DID, IB, ELISA, and LA most commonly detect antibodies to the gp43 antigen which is specific for *P. brasiliensis* complex, thus, they do not detect *P. lutzii* infection or some *P. brasiliensis* strains where the gp43 antigen is not expressed [54,56,107,109]. Antigen, PCR, and MALDI-TOF MS testing are not currently available outside research settings but may hold promise in the future [52,57,58,107,110,111].

## 6. Talaromycosis

*Talaromyces marneffei,* formerly known as *Penicillium marneffei*, is endemic to South and Southeast Asia [7,59,112,113,114]. The primary route of acquisition is pulmonary with subsequent hematogenous spread [112]. *Talaromyces* infection has been associated with exposure to soil in the rainy season [113]. *T. marneffei* causes infection in immunocompromised patients, classically people living with HIV, but more recently patients with other forms of immunosuppression have been reported as well [112,115,116]. Talaromycosis in AIDS presents as a disseminated illness with weight loss, fever, anemia, lymphadenopathy, hepatosplenomegaly, respiratory signs, and skin lesions [64,112,113]. Immunosuppressed patient without HIV commonly present with fever, cutaneous lesions, hepatomegaly, and lymphadenopathy, and are more likely to have bone and joint infection compared to HIV infected individuals [117].

Traditionally, diagnosis of *T. marneffei* has relied on fungal cultures from clinical specimens including bone marrow, skin biopsies, blood, sputum, BAL, and cerebrospinal fluid (CSF), but growth may take up to four weeks and reported performance has been highly variable [60,62,64,65,112,113,116]. Growth of *T. marneffei* on Sabouraud’s agar produces a characteristic soluble red pigment [117,118]. Blood cultures are only positive when Talaromycosis has progressed to advanced disease and can miss 30% and 50% of HIV infected and HIV non-infected patients respectively [62]. *T. marneffei* can also be diagnosed by visualization of intracellular and extracellular round to oval yeast cells (3–8 μm in diameter) from clinical specimens (Figure 1E) [59,112]. Cytology has been reported to have sensitivity of 46% in one small study [63].

Antigen detection is a promising field to aid in earlier diagnosis of *Talaromyces* with impressive progress in recent years [65,116,119,120,121]. A lateral flow immunochromatographic assay utilizing a monoclonal antibody, 4D1, conjugated with gold colloid as a signal generator and lined with *T*. *marneffei* cytoplasmic yeast antigen detected antigen in urine with a sensitivity of 87.9% and specificity of 100% in persons with confirmed *T. marneffei* infection by blood culture [64]. This assay was able to provide results in 20 min and did not require specialized equipment or skilled personnel [64]. Recently, a novel EIA using Mp1p antigen (a cell wall mannoprotein and important virulence factor for *T. marneffei*) has been developed [62,122,123]. In a large study of 372 HIV positive patients with culture proven (blood or other sterile body fluid) *T. marneffei* and 517 controls, the Mp1p EIA showed 86.3% sensitivity compared to 72.8% for blood culture [62]. Specificity for the EIA was 98.1% and time to test result was six hours versus 6.6 (± 3) days for blood culture [62]. Among 269 patients with paired urine and plasma samples tested by EIA, sensitivity increased to 88.8%, with a p value of <0.001 or 0.02 compared to plasma or urine alone [62]. These results will need to be duplicated in other populations, including immune suppressed persons without HIV but are obviously quite impressive. Although the test is not yet commercially available, a recent report describing two patients with HIV and talaromycosis diagnosed by the Mp1p EIA with negative blood cultures demonstrates the utility of the assay in concordance with the larger study results [124].

Antibody assays have been developed for diagnosis for talaromycosis; however, their clinical utility has been limited by cross reactivity, varied sensitivity and specificity, and inability of patients within the most affected populations to form antibodies due to underlying immune deficits [65,115,116,120,125]. Several traditional PCR methodologies have shown promise to detect *T. marneffei* infection but are not commercially available and are limited by variable performance [126,127,128,129]. mNGS has been used to diagnose *T. marneffei* in a HIV negative patient with disseminated disease including skin, bone marrow, CSF, and BAL involvement but its performance characteristics for talaromycosis are unknown [130].

## 7. Conclusions

Diagnosis of pulmonary infections due to endemic fungi can be challenging, as they frequently mimic other diseases. The areas of endemicity for many of these fungi are expanding, with climate change likely playing a role—this further complicates rapid diagnosis as knowledge about these infections may not be as high outside of their traditional endemic areas. Diagnosis is frequently delayed which can lead to unnecessary antibiotic exposure, repeated hospitalizations, and increased morbidity and mortality. While diagnosis has traditionally relied on culture or visualization of fungi, time to test results with culture, and insensitivity of visualization tests can limit utility. Thus, combined approaches are often necessary and rapid diagnosis frequently relies on antigen testing, with antibody testing also having a role in some cases. Cross-reactivity is a common limitation for both antibody and antigen testing but varies by the specific test and fungus. Important advances in antigen testing for talaromycosis and histoplasmosis have occurred in recent years. Molecular testing including mNGS may have a role going forward, as may MALDI-TOF MS (for identification of culture isolates), however further studies and logistical streamlining will be needed before wider adoption can be a consideration. It is important for physicians to be aware of the pulmonary manifestations of endemic fungi, as well as the changing epidemiology for many of these infections. Despite advances in diagnostic testing, a test never sent is of zero utility. A physician’s knowledge is the first step in proper diagnosis of these infections and endemic mycoses should be considered among the causes of pulmonary infections, particularly when a patient with a putative diagnosis of bacterial pneumonia does not respond to antibacterial therapy.

## Figures and Tables

**Table 1:** Signs, symptoms, imaging and lab findings, and epidemiology of pulmonary infections due to endemic mycoses *.

	Clinical Presentation	Imaging	Laboratory	Epidemiology
**Histoplasmosis**				
Acute Pulmonary Histoplasmosis	Fevers, chills, malaise, dyspnea ranging from self-limited illness to ARDS Painful compressive adenopathy. Rheumatologic manifestations	Diffuse patchy opacities Hilar/mediastinal adenopathy. Miliary pattern in severe and disseminated disease	Pancytopenia in disseminated disease Narrow based budding ovoid *Histoplasma* yeast, 2–4 μM in diameter on pathology	Central and Eastern North America, much of Central and South America, Sub-Saharan Africa, large portions of Southeast Asia, small areas of Australia and Europe
Subacute pulmonary Histoplasmosis	Mild respiratory and constitutional symptoms, >1 month	Hilar and mediastinal Adenopathy Focal/patchy airspace disease	Pancytopenia in disseminated disease Narrow based budding ovoid *Histoplasma* yeast, 2–4 μM in diameter on pathology	
Chronic Pulmonary Histoplasmosis	Fever, night sweats, weight loss, cough, shortness, chest pain of breath, >3 months	Patchy infiltrates, cavities that may enlarge over time Calcified lymph nodes. Typically no mediastinal lymphadenopathy	Narrow based budding ovoid *Histoplasma* yeast, 2–4 μM in diameter on pathology	
Blastomycosis	Acute: fevers, chills, productive cough with or without sputum production severe cases can develop ARDS. Subacute/chronic: 2–6 months: fever, night sweats, cough, hemoptysis, weight loss	Consolidation Mass like infiltrates Miliary pattern Nodules Reticular infiltrates Often no mediastinal lymphadenopathy Chronic disease typically upper lobe predominant	Broad-based budding yeast on pathology	Midwestern United States and Eastern North America, much of Africa and India
Coccidiomycosis	Fatigue, cough, fever, dyspnea, night sweats, myalgias, symptoms onset 1–3 weeks after exposure Rheumatologic phenomena	Lobar consolidation (more common), Nodular opacities Mediastinal, hilar, and/or paratracheal adenopathy Pleural effusion (typically unilateral) Cavities Miliary pattern in immunosuppressed Migratory (phantom) infiltrates	Peripheral eosinophilia Spherules range in diameter from 10 to 200 μm, are filled with 2–5 μm diameter endospores Endospores may be outside of spherules and misidentified as other fungi	*C immitis*: Central California, Washington *C posadasii*: South and Central America, Mexico, Arizona, Texas, Utah
Paracoccidiomycosis	Acute/subacute: fever, weight loss, lymphadenopathy, signs of disseminated disease Chronic/adult: cough, dyspnea, weight loss, anorexia	Reticular, nodular, interstitial or mixed opacities. Referred to as ‘bat’ or ‘butterfy’ wing in median zone Nodules Cavities. Interlobular septal thickening	Characteristic yeast resembling “pilot wheel” or “Mickey mouse” head on pathology	Parts of Central and South America, most commonly in Brazil
Talaromycosis	Fever, weight loss, cutaneous lesions, hepatosplenomegaly, lymphadenopathy, respiratory signs (both in HIV positive and HIV negative individuals) Arthritis, spondylodiskitis, osteomyelitis (more common in HIV negative individuals)	Patchy exudates Nodular infiltrates Pleural effusions Cavitary lesions Miliary pattern	Anemia, thrombocytopenia, elevated liver function tests	South and Southeast Asia

ARDS: Acute respiratory distress syndrome. * Details from this table was obtained from references cited throughout the manuscript. Laboratory or imaging findings described are typical or unique and are not meant to be all-inclusive. In cases where no notable laboratory lab abnormalities are common, none were included.

**Table 2:** Comparison of diagnostic tests utilized in the diagnosis of pulmonary infections due to endemic mycosis.

Diagnostic Test	Sensitivity	Specificity	Strengths	Limitations
***Histoplasma***				
Sputum/BAL Culture [10,11,15,16,19,20]	15–84%	Inadequate data but presumed ~100% in most studies based on reference standard definitions	More useful in SPH and CPH	Slow growth, 4–8 weeks Less useful in APH
Cytopathologic examination [8,16,17,19,20]	9–50%	Inadequate data available, generally considered fairly specific but presence of *Histoplasma* in tissue may indicate past rather than current infection. May also be misidentified	Rapid results (hours) More likely to be positive in SPH and CPH	Sensitivity and specificity vary based on pathologist experience Requires invasive procedures Less useful in pulmonary disease without dissemination.
Serum Antigen [13,19,21,22,23]	30–87%	98%	Fast results (days) Improving availability Most useful in APH	Cross reacts with other fungi Less useful in SPH and CPH
Urine Antigen [8,13,15,17,19,21,24]	40–95%	95–99%	Fast results (days) Improving availability Most useful in APH	Cross reacts with other fungi Less useful in SPH and CPH
Antibody [8,9,10,13,14]	40–95%	91%	Fast results (days) More useful in SPH and CPH	Take 4–8 weeks to develop antibodies Can be negative in immunocompromised individuals Cross reacts with other endemic mycoses
***Blastomyces***				
Sputum/ BAL Culture [1,25,26,27,28]	66–90%	Inadequate data but presumed ~100% in most studies based on reference standard definitions.	Gold Standard for diagnosis Commercial DNA (AccuProbe; GenProbe Inc., San Diego, CA) testing can provide rapid results once there is sufficient growth	Slow growth, up to 5 weeks Diagnostic yield varies based on site DNA probe can cross react with *Paracoccidioides*
Histologic or Cytopathologic examination [26,27,28,29,30]	38–93%	Inadequate data, generally considered highly specific but misidentification may occur Presence of *Blastomyces* in tissue typically indicates active infection	Rapid results (hours)	Sensitivity varies based on pathologist experience Atypical forms of *B. dermatitidis* may require special stains
Potassium hydroxide smear [25,27,28]	48–90%	No data available, generally considered highly specific but false positives possible	Rapid results	Varied sensitivity
Serum EIA Antigen [1,25,29,31,32]	36–82%	99% compared to non-fungal infections or healthy controls but 95.6 cross-reactivity with 90 cases of histoplasmosis	EDTA heat treatment improves sensitivity	Cross reacts with other fungi Only available at reference labs
Urine EIA Antigen [1,27,29,31,32,33]	76–93%	79–99%	Can be utilized to monitor response to treatment	Cross reacts with other fungi Only available at reference labs
Antibody testing via Complement fixation [28,34,35]	16–77%	30–100%	Fast results (days)	Difficult to perform, variable performance
Antibody testing via Immunodiffusion [28,29,34,36]	32–80%	100% in one study, possibility for cross-reaction remains	Fast results (days)	Can be negative in immunocompromised patients
Antibody testing via EIA (BAD-1) [33,36]	88%	94–99%	Low rate of cross reactivity Increased sensitivity when combined with antigen testing	May be negative early in infection and in immunocompromised individuals Not commercially available
***Coccidioides***				
Culture [37]	56–60%	100%	Grows well on most media in 2–7 days, specificity	Biohazard to laboratory staff
Histologic or cytopathologic examination [37,38]	22–55%	99.6%	Rapid results	Requires invasive procedures Endospores may be mistaken for *Histoplasma*, *Blastomyces* or *Cryptococcus*
Serum Antigen [39,40]	28–73%	90–100%	Most useful in immunocompromised and severe disease	Cross reactivity with *Histoplasma* and *Blastomyces*
Urine Antigen [39,41,42]	50–71%	90–98%	Most useful in immunocompromised and severe disease	Cross reactivity with *Histoplasma* and *Blastomyces*
Immunodiffusion Antibody Assays (IDTP and IDCF) [42,43]	60.2–71%	98.8%	Quantitative Titers correlated to disease severity and can monitor treatment response Commonly used as confirmatory test	Only available at reference labs Less useful in immunosuppressed patients May be negative early in disease
EIA Antibody Assay [40,43,44,45,46,47,48,49]	83–100%	75–98.5%	Commercially available Faster results	Needs confirmatory testing Not quantitative IgM cross reacts with other mycoses Less useful in immunosuppressed patients May be negative early in disease
Skin testing (Spherusol) [50]	>98%	>98% for prior exposure	Negative test may mean *Coccidioides* infection less likely	Only indicates prior exposure, unclear role in active infection
***Paracoccidioides***				
Culture [51]	25–44%	100%	Specificity	Requires 2–4 weeks to grow, infrequently used
Histologic or Cytopathologic examination [52,53]	55–97%	Presumed highly specific but inadequate data and mis-classification possible	Gold standard test, results in hours-days	Requires invasive procedures
Double Immunodiffusion Antibody Assay [52,53,54,55,56]	80−90%	>90%, inadequate data.	Most commonly utilized antibody test	Cross reactivity with other fungi Less useful for diagnosis of *P. lutzii*
ELISA Antibody Assay [52,57,58]	95.7%	85–100%	Simple to perform Fast results Antibodies can be detected at low concentrations	Cross reacts with other fungi Requires confirmatory DID Ab Less useful for diagnosis of *P. lutzii*
Latex Agglutination antibody testing [56]	69.5–84.3%	81.1%	Simple to perform	Poor reproducibility Limited availability Less useful for diagnosis of *P. lutzii*
***Talaromyces***				
Blood culture [59,60,61,62]	72.8–83%	100%	Gold standard Highly specific May culture other sterile sites as well	Takes up to 4 weeks to grow More likely to be positive in late stages of infection
Sputum culture [59,60,63]	11–34%	Inadequate data, presumed highly specific	Highly specific	Takes up to 4 weeks to grow
Culture from other tissues [59,60,61]		Inadequate data, presumed highly specific	High specificity, for some tissues, high sensitivity	
-Skin	−6–90%			-Yield only accurate if the area is involved, slow growth
-Bone Marrow	−17–100%			-Painful, variable sensitivity-Invasive, variable sensitivity
-Lymph node	−34–100%			-Invasive, small numbers studied
-Cerebrospinal fluid	−15%			-Invasive, small numbers studied
-Palatal/pharynx papule	−10%			-Painful, small numbers studied
-Liver	−5%			-Invasive, small numbers studied
-Pleural fluid	−5%			-Invasive, small numbers studied
Cytology [63]	46%	Inadequate data, presumed highly specific but misidentification may occur	Specificity	Small numbers in studies, requires invasive procedures in most cases
Lateral flow immunochromatographic antigen assay (4D1) [64]	87.9%	100%	Rapid results Easy to perform	Not commercially available Urine testing only
Antigen via EIA (Mp1p antigen) [62]	86.3%	98.1%	Rapid results Sensitivity further increased when both urine and serum tested	Not commercially available
Mab 4D1 inhibitory ELISA antigen assay [65]	100%	100%	Low cross reactivity Can be utilized on serum	Only tested on small sample size (*n* = 45), results need confirmation

Abbreviations: APH = acute pulmonary histoplasmosis, SPH = subacute pulmonary histoplasmosis, CPH = subacute pulmonary histoplasmosis, IDTP = immunodiffusion tube precipitin, IDCF = immunodiffusion complement fixation, DID = double immunodiffusion, EIA = enzyme immunoassay, ELISA = enzyme linked immunosorbent assay, Ab = antibody.
